# Preface to ‘Microwave science in sustainability’

**DOI:** 10.1098/rsta.2024.0075

**Published:** 2025-05-22

**Authors:** Daniel R. Slocombe, Adrian Porch

**Affiliations:** ^1^ Department of Engineering, Cardiff University, Cardiff, UK

**Keywords:** microwaves, radio frequency, sustainability

The microwave region of the electromagnetic spectrum has long been a subject of scientific curiosity, technological innovation and public intrigue. While many people associate microwaves primarily with their kitchen appliances, the reality is that microwaves underpin a vast array of scientific and industrial innovations, from satellite communications and quantum technologies to clean energy production and sustainable materials processing.

The rapid advances in microwave science, particularly in relation to sustainability and energy efficiency, form the core theme of this special issue of the *Philosophical Transactions of the Royal Society*. The interdisciplinary nature of microwave research means that its impact is felt across chemistry, physics, materials science and engineering, with profound implications for the future of energy, communications, waste management and recycling. The articles collected in this issue are drawn from presentations at a recent Royal Society discussion meeting that brought together leading experts across these disciplines to address fundamental questions and explore transformative new applications of microwave technology.

A key motivation for this special issue is the recognition that microwave-driven processes have the potential to reshape our approach to some of the most pressing global challenges. For example, one particularly promising area is the development of advanced battery materials, where microwaves are enabling breakthroughs in the synthesis of high-quality battery electrodes. Recent studies have shown that microwave-assisted synthesis can dramatically enhance their performance, achieving nearly 100% theoretical capacity while extending their lifespan, thereby offering a sustainable and efficient energy storage solution.

The role of microwaves in energy generation extends beyond batteries to the production of clean hydrogen and the recycling of plastic waste. Recent research has demonstrated that microwave fields, in combination with iron-based catalysts, can break down plastic waste into valuable carbon nanotubes and clean hydrogen while minimizing undesirable by-products like methane and carbon dioxide. This dual benefit—addressing both waste management and the hydrogen economy—presents an exciting avenue for sustainable fuel production. In parallel, microwave-driven catalysis has shown the potential to extract hydrogen from water at significantly lower temperatures than traditional electrolysis, offering a more energy-efficient route to hydrogen generation.

Beyond their impact on energy and sustainability, microwaves are also driving advancements in quantum computing and next-generation communication networks. The discovery of room-temperature masers has opened new possibilities for ultra-sensitive microwave detection and quantum information processing. Simultaneously, innovations in microwave laser technology are fuelling discussions on the feasibility of 6G communication networks, raising important questions about efficiency, security, and global connectivity. These advances highlight the expanding role of microwaves across a broad spectrum of technologies, underscoring their importance in shaping the future of science and industry.

Despite these promising developments, significant scientific challenges remain. The fundamental mechanisms underpinning microwave-driven reactions are still a subject of debate within the scientific community. Heated discussions have emerged over the true cause of microwave effects, with some researchers arguing that unique microwave-specific interactions are responsible, while others contend that observed phenomena can be explained by conventional thermal effects. This issue features contributions that offer experimental insights and perspectives on one of the most controversial questions in microwave science.

As society moves toward a future defined by sustainability and net-zero ambitions, it is important to establish a coherent roadmap for microwave science that aligns with these goals. The discussions presented in this issue aim to break down interdisciplinary barriers, fostering new collaborations and highlighting shared challenges and opportunities. By doing so, we hope to catalyse further innovation in microwave technologies that have the potential to drive meaningful change in energy, materials and communications.

This issue of *Philosophical Transactions of the Royal Society* provides an overview of the latest advancements in microwave science and their implications for a sustainable future. We invite readers from across the scientific spectrum to engage with these discussions, consider the emerging opportunities in their own fields and contribute to shaping the next generation of microwave-driven innovations.

## Data Availability

This article has no additional data.

